# Missed diagnosis of stroke in the emergency department: a cross-sectional analysis of a large population-based sample

**DOI:** 10.1515/dx-2013-0038

**Published:** 2014-04-03

**Authors:** David E. Newman-Toker, Ernest Moy, Ernest Valente, Rosanna Coffey, Anika L. Hines

**Affiliations:** Department of Neurology, The Johns Hopkins University School of Medicine, Baltimore, MD, USA, Phone: +410-502-6270, Fax: +410-502-6265; Agency for Healthcare Research and Quality, Rockville, MD, USA; Blue Cross Blue Shield of Minnesota, Eagan, MN, USA; Truven Health Analytics, Bethesda, MD, USA; Truven Health Analytics, Bethesda, MD, USA

**Keywords:** cerebrovascular disorders, diagnostic errors, dizziness, emergency medical services, headache, vertigo

## Abstract

**Background:**

Some cerebrovascular events are not diagnosed promptly, potentially resulting in death or disability from missed treatments. We sought to estimate the frequency of missed stroke and examine associations with patient, emergency department (ED), and hospital characteristics.

**Methods:**

Cross-sectional analysis using linked inpatient discharge and ED visit records from the 2009 Healthcare Cost and Utilization Project State Inpatient Databases and 2008–2009 State ED Databases across nine US states. We identified adult patients admitted for stroke with a treat-and-release ED visit in the prior 30 days, considering those given a non-cerebrovascular diagnosis as probable (benign headache or dizziness diagnosis) or potential (any other diagnosis) missed strokes.

**Results:**

There were 23,809 potential and 2243 probable missed strokes representing 12.7% and 1.2% of stroke admissions, respectively. Missed hemorrhages (n = 406) were linked to headache while missed ischemic strokes (n = 1435) and transient ischemic attacks (n = 402) were linked to headache or dizziness. Odds of a probable misdiagnosis were lower among men (OR 0.75), older individuals (18–44 years [base]; 45–64:OR 0.43; 65–74:OR 0.28; ≥ 75:OR 0.19), and Medicare (OR 0.66) or Medicaid (OR 0.70) recipients compared to privately insured patients. Odds were higher among Blacks (OR 1.18), Asian/Pacific Islanders (OR 1.29), and Hispanics (OR 1.30). Odds were higher in non-teaching hospitals (OR 1.45) and low-volume hospitals (OR 1.57).

**Conclusions:**

We estimate 15,000–165,000 misdiagnosed cerebrovascular events annually in US EDs, disproportionately presenting with headache or dizziness. Physicians evaluating these symptoms should be particularly attuned to the possibility of stroke in younger, female, and non-White patients.

## Introduction

Misdiagnosis, in general, may account for 40,000–80,000 preventable deaths annually in US hospitals [1] and probably a comparable amount of disability [2]. Physician-reported errors [3] and closed malpractice claims [4] indicate that stroke is among the most common dangerous missed diagnoses. One study found preventable deaths from stroke are attributed to diagnostic error over 30 times more often than deaths from myocardial infarction [5]. A better understanding of factors that predispose to stroke misdiagnosis could help spur interventions to reduce them.

Stroke, including intracranial hemorrhage, affects about 800,000 annually in the United States (US), costing over $ 40 billion [6]. Another 200,000–500,000 suffer a transient ischemic attack (TIA), a harbinger of impending stroke [6]. Stroke is a leading cause of serious disability and death in the US [7] and worldwide [8]. Early treatment improves stroke outcomes and lowers recurrent stroke risk by as much as 80% [9], so timely diagnosis is probably important. A systematic review estimated that about 9% of all strokes are not recognized at first medical contact [10].

Missed opportunities to diagnose a cerebrovascular cause for symptoms include missed stroke [11], missed TIA [12], and missed sentinel headaches from intracranial aneurysms [13]. Preventable harm may result from missed opportunities for acute treatment [14] or management to avert a second, more serious stroke or complications of the initial stroke [15]. Missed TIA and minor stroke (infarction without significant disability) or sentinel headache are often harbingers of major stroke within days or weeks without prompt intervention [16]. Small studies suggest outcomes are worse with missed stroke. Initial misdiagnosis in mild subarachnoid hemorrhage conveys a three-fold greater odds of death or severe disability at 1 year (OR 3.1; 95% CI 1.2–7.7) [13]. Mortality may be up to eight-fold greater (40% vs. 5%, p<0.001) for patients with misdiagnosed cerebellar infarctions [17].

Not all strokes are obvious clinically. Presenting symptoms that are nonspecific (e.g., dizziness [18]), mild (e.g., headache without mental status change [13]), or transient (e.g., temporary numbness [12]) are more likely to be misdiagnosed [10]. Traditional stroke symptoms such as hemiplegia are rarely missed, but “non-traditional” stroke symptoms increase the odds of misdiagnosis 43-fold [19]. Less is known about demographic and healthcare system determinants of stroke misdiagnosis. Younger patients [11, 20], women [21], minorities [22], and those triaged to lower acuity care or seen in non-teaching hospitals [23] may be at higher risk.

We sought to assess the risk and determinants of probable missed stroke in the ED on the population level, using linked ED and inpatient discharge data. Our aims were to: (1) estimate the likelihood of missed stroke diagnosis; (2) measure associations with patient, hospital, and ED visit characteristics; and (3) calculate the odds of missed stroke by those attributes.

## Materials and methods

### Study design and sample

#### Misdiagnosis concept

Definitions and standards for determining diagnostic error vary [24]. We defined a *diagnostic error* as a diagnosis that is “missed, wrong, or delayed, as detected by some subsequent definitive test or fnding” [25]. We used hospital admission with a discharge diagnosis of stroke as the “subsequent definitive test” and looked back in time from these “index” admissions for patients whose stroke-like symptoms were probably missed at a recent healthcare encounter in the ED. We used the terms ‘misdiagnosis’ and ‘diagnostic error’ interchangeably and did not distinguish among “missed,” “wrong,” or “delayed” diagnoses, as these distinctions are acknowledged to have little value in the study of diagnostic error [24]. Since we lack granular clinical data, these terms are neutral as to whether an error in the diagnostic process was made, the misdiagnosis was preventable, or any harm resulted [24].

#### Data

We conducted a retrospective, cross-sectional analysis of probable missed strokes using linked inpatient discharge records and ED visit records using Healthcare Cost and Utilization Project (HCUP) data from nine states with linkable records for the years 2008–2009 (for a detailed description of data sources, see Supplemental Data, Appendix 1, which accompanies the article at http://www.degruyter.com/view/j/dx.2014.1.issue-2/issue-files/dx.2014.1.issue-2).

### Outcomes

#### Outcome measures: potential and probable missed diagnoses

A *potentially missed stroke diagnosis* was defined as a patient with an inpatient hospital admission for stroke preceded by a treat-and-release ED visit in the prior 30 days diagnosed as anything other than a cerebrovascular diagnosis. Patients given a cerebrovascular diagnosis at a treat-and-release visit were considered *correctly diagnosed ‘bouncebacks’* if their ED discharge was followed by a stroke read-mission within 30 days. These potential errors represent a plausible upper boundary on the frequency of stroke misdiagnosis or mismanagement resulting in hospital readmission.

We defined *probable misdiagnoses* as the subset of potentially missed strokes with dizziness or headache-related diagnoses at the treat-and-release visit, since these are symptoms commonly caused by stroke but frequently misdiagnosed (e.g., benign dizziness, headaches) [11, 13, 18]. We used symptoms generally believed unrelated to stroke (e.g., back pain, abdominal pain) as control cases for comparison (i.e., likely incidental to the subsequent stroke admission) since these symptoms are rarely primary manifestations of acute stroke.

#### Analytic measures: demographic, facility, workflow, and visit characteristics

We identified patient characteristics of sex, age, race/ethnicity, payer, household income, and medical co-morbidities [26]. Hospital characteristics included region, population size of the area, ownership, and teaching status. Workflow and visit characteristics included annual volume statistics (inpatient occupancy, total ED visits, average admission fraction) and visit characteristics for the initial treat-and-release visit (weekend vs. weekday, ED crowding that day, ED admission fraction that day, and whether the patient left the ED against medical advice).

### Analysis

We examined adult (≥ 18 years old at the admission date) stroke admissions via the ED in calendar year 2009. We analyzed patients < 18 years separately. We assessed the 20 most common diagnostic groups from ED visits with potential misdiagnoses (see Supplemental Data, Appendix 1 for a detailed description of diagnosis groupings). To test our hypothesis regarding the association between symptoms and missed stroke, we performed a *visit-level* analysis, calculating the frequency and distribution of diagnoses (observed) relative to a reference ED population (expected). To examine the association of a probable mis-diagnosis with patient and hospital characteristics, we performed a *person-level* analysis, allowing only a single admission and single ED visit per patient. For person-level multivariate analyses, we used generalized estimation equation (GEE) models [27] to analyze the odds of a probable stroke misdiagnosis among adult patients admitted for stroke (with or without prior treat-and-release ED visits), controlling for patient and facility characteristics.

We conducted several validation checks to ensure coherence between our misdiagnosis construct and the results we report. First, we compared the distribution of ED diagnoses in those with potential misdiagnoses to a general ED sample of treat-and-release visits, drawn from the same national sample as our cases and controls (see Supplemental Data, Appendix 1 for details). We anticipated that the relative distribution of diagnoses would be skewed toward dizziness and headaches (known to be associated with stroke) and away from back or abdominal pain (known to not be associated with stroke). Second, we assessed the temporal profile of probable missed diagnoses within the 30-day window prior to index stroke admission. We hypothesized readmissions would be disproportionate in the first few days after the initial ED treat-and-release and diminish over time, as shown previously for major stroke after TIA and minor stroke [16]. Third, we analyzed the association between stroke type (TIA, ischemic stroke, intracerebral hemorrhage, subarachnoid hemorrhage) and initial presenting symptom. We hypothesized that dizziness/vestibular diagnoses would be associated with ischemic events (TIA or stroke) and headache/migraine diagnoses would be linked to hemorrhages (of en misdiagnosed as benign headaches) and TIAs (of en misdiagnosed as migraine-related transient neurologic symptoms).

## Results

### Frequencies and distributions

**Figure 1:**
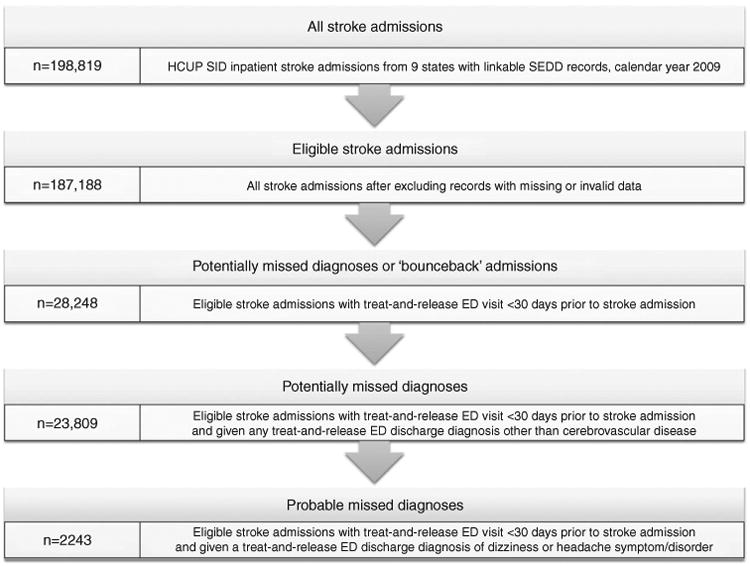
Flow diagram of study population derivation.

Figure 1 shows data flow. Of 198,819 stroke admissions in 2009, 187,188 (94%) had complete and administratively correct records for analysis. Of these, 28,248 (15.1%) were potentially missed *or* ‘bouncebacks’ and 2 3,809 (12.7%) were potentially missed strokes (i.e., discharged from the initial ED visit with a non-cerebrovascular diagnosis).

**Figure 2:**
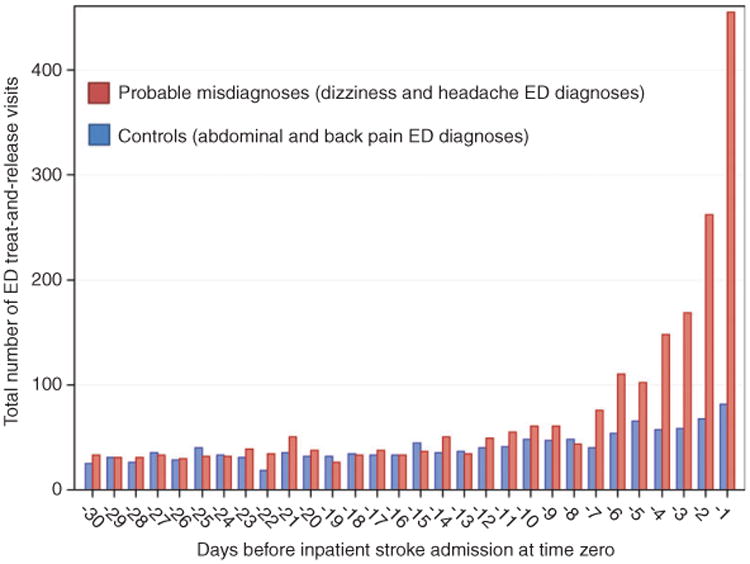
Temporal profile analysis of initial ED treat-and-release visits for probable misdiagnoses (dizziness/headaches) versus controls (back problems/abdominal pain) in the 30 days prior to an index stroke admission. ED, emergency department.

The 20 most common reasons for the initial ED visit among those potentially missed or ‘bouncebacks’ are shown in Table 1. Most potential misdiagnoses involved none of the targeted ‘case’ or ‘control’ diagnoses, but these ‘other’ diagnoses were, in aggregate, under-represented relative to an average ED treat-and-release population (Table 2). Patients with an initial cerebrovascular diagnosis were the most likely to return for a stroke admission (Table 2). As hypothesized, those released with dizziness or headache (probable misdiagnoses) were over-represented, while back pain or abdominal pain diagnoses (control conditions) were under-represented (Table 2; Supplemental Data, Appendix 2 contains data for patients < 18 years). Among potential misdiagnoses, 10.4% involved a dizziness or headache diagnosis (i.e., probable misdiagnoses), representing 1.2% of all stroke admissions. Dizziness was associated predominantly with missed ischemic strokes, whereas headache was associated with both ischemic and hemorrhagic forms of stroke (Table 3). Temporal profile analysis (Figure 2; Supplemental Data, Appendix 3) indicated that treat-and-release ED visits for probable misdiagnoses were clustered in the few days prior to the hospital admission for stroke, compatible with the known biology of TIA and minor stroke preceding major, disabling stroke [16]. This is in contrast to controls with back problems or abdominal pain whose visits were evenly distributed throughout the 30-day window (Figure 2). Note also that among other diagnoses (non-stroke, non-dizziness/headache, non-back/abdominal pain), a similar clustering was observed (Supplemental Data, Appendix 3), arguing that some of these potential misdiagnoses, which outnumbered the dizziness/headache diagnoses, were also likely true misdiagnoses.

### Multivariate analysis

Multivariate analysis of patient and hospital characteristics associated with probable missed stroke diagnoses is shown in Table 4.

#### Patient characteristics

Males had 25% lower odds of misdiagnosis (OR 0.75; p < 0.001). Increasing age was associated with decreasing odds of missed stroke diagnosis; compared to age 18–44, odds for other age groups were: 45–64: OR 0.43; 65–74: OR 0.28; 75 +: OR 0.19 (all p < 0.001). The proportion of probable missed strokes in each age group was: 3.98% (18–44), 1.70% (45–64), 0.91% (65–74), 0.59% (75 +). Compared to non-Hispanic White patients, others had higher odds of a missed stroke diagnosis: Black (OR 1.18; p = 0.02), Asian/Pacific Islander (OR 1.29; p = 0.02), and Hispanic (OR 1.30; p < 0.001).

Privately-insured patients had the highest odds of a missed stroke. In comparison, lower odds of missed stroke were demonstrated by patients with Medicare at 34% (OR 0. 6 6; p < 0.001), Medicaid at 30% (OR 0. 7 0; p < 0.001), and “other payer” at 37% (OR 0.63; p = 0.003). Patient income level had no incremental association with missed stroke. The presence of medical comorbidities was associated with reduced odds of stroke misdiagnosis. Each additional comorbidity decreased the incremental odds of a missed diagnosis by 7% (p = 0.02).

#### Hospital characteristics

Relative to large metropolitan areas, patients in facilities located in small metropolitan areas demonstrated 23% lower odds of a missed stroke (OR 0.77; p = 0.003). Large metropolitan, micropolitan, and rural locations did not differ. Non-teaching hospitals demonstrated 45% higher odds of missed stroke than teaching hospitals (OR 1.45; p < 0.001). Compared to hospitals with high ED volume, hospitals with low volume demonstrated 57% higher odds of missed stroke (OR 1.57; p = 0.007). The region of the country where the facility was located and facility ownership were not associated with stroke misdiagnosis.

#### Hospital workflow and ED visit characteristics

The overall facility inpatient occupancy rate was not associated with the likelihood of misdiagnosis, but the overall annual admission rate from the ED and the ED admission rate on the day of the ED visit were both inversely related to missed stroke (i.e., higher admission rates correlated with lower odds of misdiagnosis). By contrast, relative ED crowding (ratio of ED visits that day to the maximum one-day ED volume in that year) was not associated with missed stroke. Visits taking place on weekdays had 11% higher odds of a missed stroke than those occurring on weekends (OR 1.11; p < 0.04). Compared to those *not* discharged against medical advice, 81 individuals (3.7%) who left the ED against medical advice demonstrated nearly three times the odds of missed stroke (OR 2.94; p < 0.001).

## Discussion

### Overall missed stroke estimates

To our knowledge, this is the first large-scale, multi-regional study to examine clinical, hospital, and visit-related predictors of missed diagnosis of stroke in US EDs. Using 30-day readmission for stroke, we found that 12.7% of stroke admissions were potentially misdiagnosed. The top two potential misdiagnoses preceding a stroke read-mission were headache and dizziness. This finding is consonant with previous studies indicating these particular symptoms are often associated with missed strokes [10, 14, 18, 20, 23, 28, 29]. These probable misdiagnoses accounted for 1.2% of all strokes admissions and 10.4% of potential misdiagnoses. Thus, we estimate that the overall prevalence of misdiagnosis followed by delayed hospital admission after ED discharge ranges between 1.2% and 12.7% of all stroke admissions. With 1.3 million new or recurrent cerebrovascular events per year in the US [6], this corresponds to approximately 15,000 to 165,000 missed strokes and TIAs annually. Most missed strokes were ischemic rather than hemorrhagic (ratio 5:1), roughly in proportion to their population prevalence [6]. The impact and pre-ventability of these misdiagnoses remains unknown.

Previous estimates of ED stroke misdiagnosis rates range from as low as 1.2% (considering admitted patients at an academic teaching hospital but not those discharged from the ED [30]) to as high as 50% (for predominantly Hispanic patients presenting with isolated dizziness or vertigo to non-teaching hospital EDs in Nueces County, Texas [18]). Our findings accord with the results of a systematic review (9.2% missed among more than 5000 patients with stroke from seven studies [10]) and the only population-based study of all-stroke misdiagnosis identified in that review (8.5% missed among 1800 patients with stroke in rural Texas [31]).

The current study's 1.2% estimate for *probable misdiagnosis* is conservative. It does not count those who suffered no major adverse event (i.e., readmission); those with an erroneous diagnosis other than dizziness or headache (e.g., “gastroenteritis” diagnosed at the treat-and-release visit, with cerebellar infarction identified during the subsequent visit and hospitalization [11]); those with stroke at the initial ED visit admitted to a non-stroke clinical service with the wrong tentative diagnosis; those whose causally-related stroke occurred more than 30 days after an initial ED visit (e.g., carotid artery dissection presenting with headache, followed by a stroke at day 31 [32]); or those who suffered fatal stroke after ED treat-and-release. Although some initial ED visits we counted as probable misdiagnoses might have been coincidental, the fact that most were clustered in the few days before the stroke admission suggests otherwise. This temporal pattern with the highest risk in the first 48 h after discharge matches the time window of greatest risk for major stroke after TIA and minor stroke, where about three-fourths of the 30-day risk occurs in the first week [16]. It is further corroborated by the fact that this pattern was not observed for control subjects (Figure 2).

### Symptom characteristics

As we hypothesized, dizziness and headache visits were over-represented while back and abdominal pain visits were under-represented among subsequent stroke admissions. The odds of a dizziness discharge preceding a stroke admission were 2.4-times expected, and headache discharges were 1.9-times expected. This is not surprising, given prior smaller-scale studies suggesting that presentations with isolated dizziness and headaches caused by stroke are more often misdiagnosed. For example, population-based studies in Texas found ischemic stroke mis-diagnosis rates of 4% for patients with motor symptoms, 35% for those with dizziness, and 50% for those with isolated dizziness (i.e., with no other neurological symptoms) [18, 31]. Aneurysmal subarachnoid hemorrhages are more likely to be misdiagnosed when they present with isolated headache (19%) versus with accompanying mental status changes (5%) [13]. Strokes presenting with only “non-traditional” symptoms are misdiagnosed frequently compared to those with “traditional” symptoms (64% vs. 4%, OR 43.4; 95% CI 15.0–125.4) [19]. Considered with our findings, these results suggest that clinical presentations with “atypical” or “mild” stroke symptoms likely have relevance at a national level and should be targeted for misdiagnosis-reduction interventions.

### Patient characteristics

Age was strongly (and inversely) associated with misdiagnosis. Those older than 75 years were 80% less likely to be misdiagnosed than those 18–44, corroborating data from small studies showing mostly young patients among those misdiagnosed [11, 20]. It is probably not surprising that stroke, generally considered a disease of the elderly, would be misdiagnosed in the young. Although missing strokes in younger patients may result from appropriate diagnostic reasoning that considers age-specific prevalence, clinical decision rules to detect stroke in dizziness [33] and headache [34] are not age dependent and may enable efficient bedside stroke detection using history and physical examination, even in patients with low baseline prevalence.

Women were at greater odds of misdiagnosis, and a greater frequency of non-classic stroke presentations could be to blame [35], as with acute coronary syndromes [36]. Women are also more likely to experience dizziness and headaches of benign cause [37–39], increasing the challenge of differentiating strokes from stroke mimics. Hispanic and non-White patients were more likely to be misdiagnosed, in keeping with prior studies of stroke [31] and myocardial infarction [40]. The causes of this racial disparity are not clear, but some evidence suggests that diagnostic stroke workups are less thorough for Hispanics [41]. Greater vigilance for stroke in women, minorities, and younger patients appears warranted.

Comorbid conditions may increase the suspicion of stroke or be associated with greater overall likelihood of admission, so it seems reasonable that lesser comorbidity was associated with greater likelihood of missed stroke. Private insurance was a risk factor for misdiagnosis relative to both Medicare and Medicaid but income was not, perhaps suggesting that private insurance was a partial surrogate for lower medical comorbidity or complexity, or that private insurance reduced the likelihood of initial admission in a nonspecific way.

### Hospital characteristics, workflow, and ED visit characteristics

Hospitals with high annual ED volume and teaching hospitals had lower odds of misdiagnosis. Whether these differences reflect greater diagnostic acumen, a lower threshold for additional diagnostic testing, or a general tendency toward admission driven by profit motive or legal conservatism remains uncertain. There was no geographic variation by hospital region but, somewhat surprisingly, hospitals located in large metropolitan areas were more likely to miss stroke than those in small metropolitan areas. The source of this variation is unclear, but it matches that found in a similarly conducted analysis of HCUP data for misdiagnosis of acute myocardial infarction [40], so probably merits further investigation.

One of the largest factors identified in this study was progressively greater odds of misdiagnosis on days with lower ED admission rates – more than six-fold higher on days in the lowest ED admission rate quintile. It seems sensible that discharging a greater fraction of ED patients would be associated with a greater likelihood of misdiagnosis. Although there are many plausible explanations, one possibility could be that different ED physicians have different risk thresholds for obtaining diagnostic workups and admitting patients [42], resulting in substantial day-to-day variations in admission rates that correlate with the likelihood of misdiagnosis.

Although ED crowding as measured here was not associated with missed stroke, leaving against medical advice was associated with a 2.9-fold increase in odds of misdiagnosis. Since leaving against medical advice is associated with ED crowding [43], the impact of crowding on misdiagnosis should be explored further in future studies. The finding of greater odds of misdiagnosis on weekdays runs counter to findings from prior studies and should be interpreted with caution. Further study of hospital and physician determinants of misdiagnosis is warranted.

### Implications for diagnostic error research

The analytic methods described in this manuscript hold promise as a possible trigger tool [44] or metric for identifying and assessing the overall burden of dangerous diagnostic errors in the ED using administrative data. Although generic ED revisit analyses do not predict errors well [45], the specific symptom-disease pair approach has been used successfully to identify diagnostic errors based on ED revisit analyses [23]. Our novel approach of referencing observed (‘cases’) versus expected (‘control’) distributions of symptoms at ED visits offers two important advantages over prior methods. First, it serves as an important clinical validity check on the accuracy of results derived from billing data. Second, it offers insight into the relative risks of misdiagnosis across a range of symptoms, allowing prioritization across symptoms-disease pairs. From an epidemiologic perspective, our ‘look-back’ approach (from revisit/harm back to initial presentation) could be used to identify symptom-disease pairs that are prevalent and high-risk for misdiagnosis-related harm. These highest-impact symptom-disease pairs could then be monitored using ‘look-forward’ approaches (from initial presentation to revisit/harm) [28].

### Implications for clinical care

Reducing ED stroke misdiagnosis may not be easy. Despite the rapid increase in use of advanced neuroimaging and other expensive laboratory diagnostic tests [46], there is little evidence that this “technocentric” approach correctly diagnoses more strokes [47, 48]. Routine access to neurologists, advocated by some [49], is probably impractical. Telestroke consultation could be cost-effective for accessing expertise remotely [50]. Unfortunately, tele-consults do not directly address atypical cases most likely to be missed [19], since consultation may not be sought. Computer-based diagnostic decision support may someday help, but no systems have yet demonstrated real-world benefit in stroke diagnosis [51]. Bedside methods for identifying high-risk patients [33, 34, 52–54] offer evidence-based [17], cost-effective [55] alternatives. Efforts to disseminate these history and physical examination-based approaches outside of specialty circles [56–58], possibly assisted by emerging device-based technologies [59], could be a promising strategy to reduce misdiagnosis. Implementations should be coupled to misdiagnosis metrics similar to those used here to objectively demonstrate error reduction.

### Limitations

Limitations relate primarily to use of administrative data (without granular clinical details) and cross-sectional study design. All misdiagnoses reported here are inferred, since charts could not be directly reviewed; thus we could not control or adjust for potential confounding related to comorbidities. Administrative errors in coding may have influenced the results, and it is possible that some inpatient stroke diagnoses were incorrect. Misdiagnosed strokes not resulting in readmission were not captured. Some probable misdiagnoses may have been coincidental, and many of the ‘bounceback’ cases (stroke discharge followed by readmission) may have been unavoidable. Our cross-sectional results can only determine associations, not causation. Our categories of ‘probable’ and ‘possible’ misdiagnoses have not been previously validated with ‘gold standard’ cases where misdiagnosis status was known definitively. Unmeasured factors that could influence odds of misdiagnosis (e.g., physician training, experience, or risk aversion; ready availability of neuroimaging or neurologic consultation in the ED; stroke center certification [60]) might confound the association with some of our measured predictors (e.g., hospital type, ED admission fraction). Some known predictors of misdiagnosis (e.g., ED triage severity level [23]) could not be measured. Multivariate associations may partly reflect specific attributes of misdiagnosis related to headache and dizziness, so may not generalize completely to other clinical presentations of stroke. Harm and preventability could not be directly assessed; it is unknown how many of those misdiagnosed underwent appropriate neuroimaging. Although the sample is large (187,188 patients and 1016 hospitals), this work is based on data derived from only nine US states, does not consider non-ED (e.g., primary care) missed stroke, and might not be generalizable to other settings nationally or internationally.

## Conclusions

Although further research is needed to confirm our findings using more direct methods of diagnostic error detection (e.g., direct chart review), our present study strongly suggests stroke misdiagnosis can be measured using administrative data. Stroke misdiagnoses appear to be associated preferentially with dizziness and headache presentations. This study provides some immediate suggestions to ED physicians who are evaluating patients with these symptoms – be more attuned to the possibility of stroke in younger, female, and non-White patients. Though ‘simple’, indiscriminate use of neuroimaging will not prove an effective strategy to detect stroke in these patients [47, 48, 55]. Instead, clinicians should leverage well-studied bedside methods to identify dizziness and headache patients at high risk for stroke [33, 34, 52–54].

General policy recommendations for hospitals and other healthcare stakeholders are less clear, pending further substantive research on methods to measure misdiagnosis and strategies to reduce them in the ED. Funding agencies should support studies to develop and refine revisit analyses as a means to measure the burden of misdiagnosis in the ED, along with systematic study of disparities in misdiagnosis based on sex, age, and race/ethnicity. Without robust measurement methods, it will be impossible to assess the impact of interventions. Although many general solutions to help reduce misdiagnosis have been developed, none have yet been properly studied for their impact on patient outcomes [51]. Problem-specific solutions may hold more promise than general ones [1], and model-based economic analyses could help guide evidence-based policy development for misdiagnosis problems causing the greatest harms [55]. Future research should seek to identify targeted error-reduction interventions to reduce stroke misdiagnosis and improve patient outcomes at reasonable cost.

## Supplementary Material

Appendix 1 Table. Healthcare Cost and Utilization Project (HCUP) Clinical Classification System (CCS) Multilevel and ICD-9-CM codes used in analyses
Appendix 2 Table. Observed emergency department (ED) non-adult (age <18 years) treat-and-release visits* that were followed by a stroke admission within 30 days, compared to the expected percentages, based on all ED treat-and-release visits
Appendix 3 Figure. Temporal profile analysis of initial ED treat-and-release visits in the 30 days prior to an index stroke admission

## Figures and Tables

**Table 1:** Twenty most common reasons^a^ for adult emergency department (ED) treat-and-release visits^b^ that were followed by a stroke admission within 30 days, 2009.

	Clinical Classification Software label: description	Frequency
1	109: Acute cerebrovascular disease	4439
2	84: Headache; including migraine	1450
3	112: Transient cerebral ischemia	1339
4	239: Superficial injury; contusion	1003
5	93: Conditions associated with dizziness or vertigo	984
6	95: Other nervous system disorders	982
7	102: Nonspecific chest pain	940
8	98: Essential hypertension	775
9	252: Malaise and fatigue	768
10	259: Residual codes; unclassified	749
11	211: Other connective tissue disease	717
12	205: Spondylosis; intervertebral disc disorders; other back problems	677
13	251: Abdominal pain	647
14	159: Urinary tract infections	611
15	244: Other injuries and conditions due to external causes	509
6	232: Sprains and strains	487
17	245:Syncope	477
18	133: Other lower respiratory disease	439
19	250: Nausea and vomiting	344
20	127: Chronic obstructive pulmonary disease and bronchiectasis	341
76	94: Other ear and sense organ disorders	52
	All other codes	9518
	Total	28,248

a Also shown is category ‘94′ – the only dizziness or headache category that was not already listed in the top 20 (i.e., 84 and 93). Together, 93 and 94 make up ‘dizziness.’

b This is a visit-level analysis, so counts include multiple ED visits by a single person.

**Table 2:** Observed emergency department (ED) adult treat-and-release visits a that were followed by a stroke admission within 30 days, compared to the expected percentages based on all treat-and-release visits, 2009.

ED visit diagnosis	Observed^b^ number	Observed percent	Expected^c^ percent	Observed/expected ratio d
Correctly diagnosed ‘bounceback’ strokes				
Cerebrovascular disease (CCS = 109)	4439	15.71%	0.10%	157.10
Probable misdiagnosed strokes				
Headache (including migraine) (CCS = 84)	1450	5.13%	2.65%	1.94
Dizziness (CCS = 93; CCS = 94)	1036	3.67%	1.53%	2.40
Suspected incidental diagnoses (control conditions) among potentially misdiagnosed strokes				
Back problems (CCS = 205)	677	2.40%	3.06%	0.78
Abdominal pain (CCS = 251)	647	2.29%	4.43%	0.52
Other (all other CCS codes)	19,999	70.80%	88.23%	0.80
Total	28,248	100.00%	100.00%	

CCS, Agency for Healthcare Research and Quality Clinical Classification Software (CCS) for ICD9-CM (single-level groupings).

a This is a visit-level analysis, so counts include multiple ED visits by a single person.

b Observed: frequency and corresponding percentage of first-listed CCS code in the State Emergency Department Database visit record for potentially missed diagnoses or ‘bouncebacks’ identified in the study sample.

c Expected: proportion of ED treat-and-release visits to have identified diagnoses, based on all ED treat-and-release visits.

d Observed to expected ratio of proportions: values > 1 reflect disproportionate over-representation compared to an average ED population; values < 1 reflect disproportionate under-representation compared to an average ED population.

**Table 3:** Probable missed diagnoses of stroke (misdiagnosed as dizziness or headache) during an ED treat-and-release visit, for adult patients a later admitted for stroke, by type of stroke diagnosed during the inpatient stay, 2009.

Diagnosis at most recent ED visit for:	Inpatient diagnosis: type of stroke				Total, %

	Transient ischemic attack (TIA)	Acute ischemic stroke (AIS)	Intracerebral hemorrhage (ICH)	Subarachnoid hemorrhage (SAH)	
Dizziness n, %	184 (19%)	732 (75%)	54 (5%)	7 (1%)	977(100%)
Headache n, %	218 (17%)	703 (55%)	172 (14%)	173 (14%)	1266(100%)
Total n, %	402 (18%)	1435 (64%)	226(10%)	180 (8%)	2243 (100%)

Emergency department (ED) visits: Agency for Healthcare Research and Quality Clinical Classification Software (CCS) codes—dizziness (codes 93 [dizziness and vertigo] or 94 [other ear conditions]); headache (code 84 [headache]). Inpatient diagnoses of stroke: ICD-9-CM codes—TIA (435.x); ICH (431, 432.x); SAH (430); AIS (any other stroke code listed in Appendix 1 Table).

a This is a patient-level analysis, so counts only include the most proximate ED visit.

**Table 4:** Factors associated with a missed diagnosis of stroke (based on prior ED treat-and-release visits for dizziness or headache 30 days before stroke admission) among all patients^a^ aged > = 18 years admitted to inpatient care for stroke: generalized estimating equation (GEE) results, 2009.

Data element	Value	EST	SE	Z	p	OR	LCL	UCL
Patient characteristics								
Sex	0: Male	–0.29	0.05	–6.16	<0.001	0.75	0.68	0.82
	1: Female							
Age group, years	45–64	–0.85	0.07	–12.99	<0.001	0.43	0.38	0.49
	65–74	–1.26	0.11	–11.58	<0.001	0.28	0.23	0.35
	75 and over	–1.68	0.12	–14.56	<0.001	0.19	0.15	0.23
	18–44							
Race/ethnicity	Black	0.17	0.07	2.39	0.02	1.18	1.03	1.35
	Hispanic	0.27	0.08	3.27	<0.001	1.30	1.11	1.53
	Asian/Pacific Islander	0.25	0.11	2.30	0.02	1.29	1.04	1.60
	White							
Payer	Medicare	–0.41	0.10	–4.30	<0.001	0.66	0.55	0.80
	Medicaid	–0.36	0.09	–3.89	<0.001	0.70	0.58	0.84
	Uninsured	0.01	0.09	0.11	0.91	1.01	0.85	1.20
	Other	–0.46	0.15	–2.98	0.003	0.63	0.47	0.85
	Private insurance							
Income quartile	Lowest income < 40K	0.06	0.08	0.75	0.45	1.06	0.90	1.25
	Low income 40K to < 50K	0.05	0.08	0.65	0.52	1.05	0.90	1.23
	Moderate income 50K– < 66K Highest income ≥ 66K	0.05	0.08	0.61	0.54	1.05	0.90	1.23
Comorbidities	Range: 0 – 13	–0.07	0.02	–4.00	<0.001	n/a	n/a	n/a
Hospital characteristics								
Region	Midwest	–0.17	0.15	–1.1	0.27	0.84	0.62	1.14
	South	–0.12	0.13	–0.93	0.35	0.88	0.68	1.14
	West	–0.03	0.12	–0.24	0.81	0.97	0.77	1.22
	Northeast							
Population size	Small metropolitan	–0.26	0.09	–2.94	0.003	0.77	0.65	0.92
	Micropolitan	0.21	0.15	1.41	0.16	1.23	0.92	1.64
	Rural	0.06	0.24	0.24	0.81	1.06	0.67	1.68
	Large metropolitan							
Ownership	Public	–0.01	0.12	–0.10	0.92	0.99	0.78	1.25
	Private, for–profit	–0.22	0.12	–1.93	0.05	0.80	0.64	1.00
	Private, not-for-profit							
Teaching Status	Nonteaching	0.37	0.11	3.24	<0.001	1.45	1.16	1.82
	Teaching							
Hospital workflow (annual average)								
Inpatient occupancy rate (annual)	Low ≤ 0.5	0.00	0.13	0.03	0.98	1.00	0.78	1.29
	Moderate > 0.5, < 0.7	0.11	0.11	0.94	0.35	1.11	0.89	1.39
	High ≥ 0.7							
ED volume (annual)	Low ≤ 29,124	0.45	0.17	2.69	0.007	1.57	1.13	2.18
	Moderate 29,125–64,434	0.10	0.10	1.02	0.31	1.11	0.91	1.36
	High ≥ 64,435							
Percent admitted from ED (annual)	Low ≤ 11.82%	0.44	0.15	2.88	.004	1.55	1.15	2.09
	Moderate > 11.82%, < 19.46%	0.21	0.11	1.95	0.05	1.24	1.00	1.54
	High ≥ 19.46%							
ED visit characteristics (day of initial treat-and-release visit)								
Weekend	Monday–Friday	0.11	0.05	2.09	0.04	1.11	1.01	1.23
	Saturday–Sunday							
ED crowding on day of visit (percentile)	0–20th percentile	–0.02	0.07	–0.33	0.75	0.98	0.84	1.13
	21 – 40th percentile	0.04	0.07	0.5	0.62	1.04	0.90	1.19
	41 – 60th percentile	0.04	0.07	0.52	0.60	1.04	0.90	1.20
	61 – 80th percentile	0.08	0.07	1.18	0.24	1.08	0.95	1.23
	61 – 80th percentile							
ED admit rate on day of visit (percentile)	0 – 20th percentile	1.85	0.16	11.72	<0.001	6.34	4.66	8.63
	21 – 40th percentile	0.91	0.11	8.03	<0.001	2.48	1.99	3.10
	41 – 60th percentile	0.61	0.10	6.05	<0.001	1.85	1.51	2.25
	61 – 80th percentile	0.34	0.08	4.04	<0.001	1.40	1.19	1.66
	81 – 100th percentile							
Patient left against advice	Against medical advice	1.08	0.14	7.50	<0.001	2.94	2.22	3.89
	Not against advice							

187,188 of 198,819 trials used; number of events used = 2088 of 2243 (records with missing data excluded); exchangeable correlation structure (working correlation = 0.002); 1016 clusters (facilities). EST, estimate; SE, standard error; Z, Z score; p, probability level; OR, odds ratio; LCL, lower confidence limit; UCL, upper confidence limit.

a This is a patient-level analysis of inpatient stroke admissions, with and without a prior treat-and-release ED visit for dizziness or headache within 30 days of the stroke admission; only a single ‘initial’ ED visit (the most proximate to the ‘index’ stroke admission) is considered.
