# Supplementary material for: Missed diagnosis of stroke in the emergency department: a cross-sectional analysis of a large population-based sample
Source: Diagnosis (Berl). Author manuscript; Available in PMC 2017 Mar 22. (PMC5361750; doi:10.1515/dx-2013-0038)

Appendix 1 Table. Healthcare Cost and Utilization Project (HCUP) Clinical Classification System (CCS) Multilevel and ICD-9-CM codes used in analyses

Measure	CCS label (single level)	CCS code (level 3)	ICD-9-CM codes
Cerebrovascular disease	Acute cerebrovascular disease [109.]	7.3.1	34660 34661 34662 34663 430 431 4320 4321 4329 43301 43311 43321 43331 43381 43391 4340 43400 43401 4341 43410 43411 4349 43490 43491 436
	Intracranial hemorrhage	7.3.1.1	430 431 4320 4321 4329
	Occlusion of cerebral arteries	7.3.1.2	43301 43311 43321 43331 43381 43391 4340 43400 43401 4341 43410 43411 4349 43490 43491
	Acute, but ill-defined cerebrovascular accident	7.3.1.3	34660 34661 34662 34663 436
	Occlusion or stenosis of precerebral arteries [110.]	7.3.2	4330 4331 43310 4332 43320 4333 43330 4338 43380 4339 43390
	Other and ill-defined cerebrovascular disease [111.]	7.3.3	4370 4371 4373 4374 4375 4376 4377 4378 4379
	Transient cerebral ischemia [112.]	7.3.4	4350 4351 4352 4353 4358 4359
Dizziness	Conditions associated with dizziness or vertigo [93.]	6.8.2	38600 38601 38602 38603 38604 38610 38611 38612 38619 3862 38630 38631 38632 38633 38634 38635 38640 38641 38642 38643 38648 38650 38651 38652 38653 38654 38655 38656 38658 3868 3869 7804
	Other ear and sense organ disorders [94.]	6.8.3	38000 38001 38002 38003 38010 38011 38012 38013 38014 38015 38016 38021 38022 38023 38030 38031 38032 38039 3804 38050 38051 38052 38053 38081 38089 3809 400 38401 38409 3841 38530 38531 38532 38533 38535 38582 38583 38589 3859 38800 38801 38802 38810 38811 38812 3882 38830 38831 38832 38840 38841 38842 38843 38844 38845 3885 38860 38861 38869 38870 38871 38872 3888 3889 38900 38901 38902 38903 38904 38905 38906 38908 38910 38911 38912 38913 38914 38915 38916 38917 38918 3892 38920 38921 38922 3897 3898 3899 V412 V413 V4985 V532 V721 V7211 V7212 V7219

Headache	Headache, including migraine [84.]	6.5.1	33900 33901 33902 33903 33904 3460 34600 34601 34602 34603 3461 34610 34611 34612 34613 3462 34620 34621 34622 34623 34630 34631 34632 34633 34640 34641 34642 34643 34650 34651 34652 34653 34670 34671 34672 34673 3468 34680 34681 34682 34683 3469 34690 34691 34692 34693
		6.5.2	33905 33909 33910 33911 33912 33920 33921 33922 3393 33941 33942 33943 33944 33981 33982 33983 33984 33985 33989 7840
Back problems	Spondylosis; intervertebral disorders; other back problems [205.]	13.3.1	7210 7211 7212 7213 72141 72142 7215 7216 7217 7218 72190 72191
		13.3.2	7220 72210 72211 7222 72230 72231 72232 72239 7224 72251 72252 7226 72270 72271 72272 72273 72280 72281 72282 72283 72290 72291 72292 72293
		13.3.3	7234 7235 7236 7237 7238 7239 72400 72401 72402 72403 72409 41 7242 7243 7244 7245 7246 72470 72471 72479 7248 7249
Abdominal pain	Abdominal pain [251.]	17.1.7	7890 78900 78901 78902 78903 78904 78905 78906 78907 78909 78960 78961 78962 78963 78964 78965 78966 78967 78969 7890 78900 78901 78902 78903 78904 78905 8906 78907 78909 78960 78961 78962 78963 78964 78965 78966 78967 78969

Appendix 2. Results for Patients <18 Years of Age

Because the spectrum of clinical stroke presentations differs in children, we separately analyzed patients <18 years. There were a smaller number of relevant cases, because stroke is of low incidence in this age group. Although based on a small sample size, it appears that headache treat-and-release diagnoses are dramatically over-represented in the 30 days prior to a stroke admission, while other diagnoses are under-represented (Appendix 2 Table).

Appendix 2 Table. Observed emergency department (ED) non-adult (age <18 years) treat-and-release visits* that were followed by a stroke admission within 30 days, compared to the expected percentages, based on all ED treat-and-release visits

**ED visit diagnosis**	**Observed[a] number**	**Observed Percent**	**Expected[b] percent**	**Observed / expected ratio[c]**
Correctly diagnosed 'bounceback' strokes				
Cerebrovascular disease	18	17.48%	0.01%	2729.61
Probable misdiagnosed strokes				
Headache (including migraine)	11	10.68%	0.97%	10.97
Ear conditions (including vestibular)	**	**	1.08%	-**
Suspected incidental diagnoses (control conditions) among potentially misdiagnosed strokes				
Back problems	**	**	0.48%	**
Abdominal pain	**	**	2.83%	**
Other	68	66.02%	94.63%	0.70
Total	103	100.00%	100.00%	

Note: diagnosis shown is the first diagnosis code listed in the State Emergency Department Database visit record for potentially missed diagnoses or 'bouncebacks' identified in the study sample; diagnosis codes grouped using Agency for Healthcare Research and Quality Clinical Classification Software for ICD9-CM (multi-level groupings).

* This is a visit-level analysis, so counts include multiple ED visits by a single person.

**Indicates where cell values are <10. These should not be reported in accordance with HCUP research guidelines.

[a]Observed: frequency and corresponding percentage of first-listed CCS code in the State Emergency Department Database visit record for potentially missed diagnoses or 'bouncebacks' identified in the study sample.

[b]Expected: proportion of ED treat-and-release visits to have identified diagnoses, based on all ED treat-and-release visits.

[c]Observed to expected ratio of proportions: values >1 reflect disproportionate over-representation compared to an average ED population; values <1 reflect disproportionate under-representation compared to an average ED population.

Appendix 3. Temporal Profile Analysis for ED Visits

The temporal profile analysis for ED treat-and-release visits in the 30 days prior to an inpatient stroke admission demonstrates a clustering of early revisits in close temporal proximity to the inpatient stroke admission. This finding is suggestive of a link to missed stroke, since this is the temporal profile of major stroke following minor stroke or transient ischemic attack. The pattern is evident for those correctly diagnosed 'bouncebacks' (Figure, A), probable stroke misdiagnoses (B), and other potential misdiagnoses (C), but not for controls (B).

Appendix 3 Figure. Temporal profile analysis of initial ED treat-and-release visits in the 30 days prior to an index stroke admission

Abbreviations: CVA – cerebrovascular accident; ED – emergency department; IP – inpatient

A. Stroke treat-and-release diagnoses (correctly diagnosed 'bouncebacks')

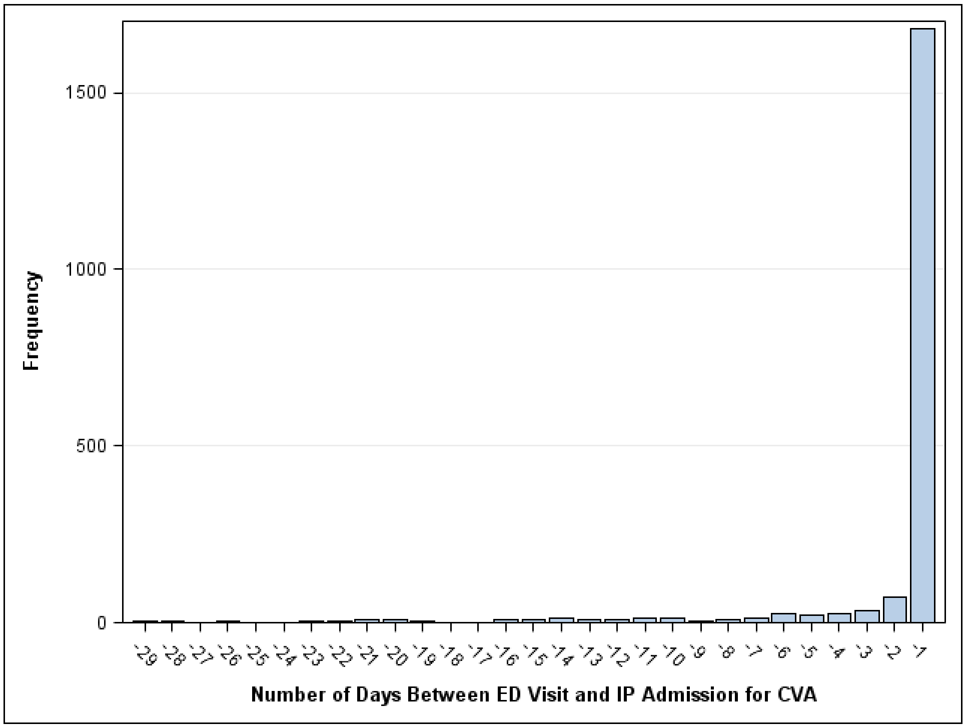

B. Probable misdiagnoses (dizziness/headache) and controls (back/abdominal pain)

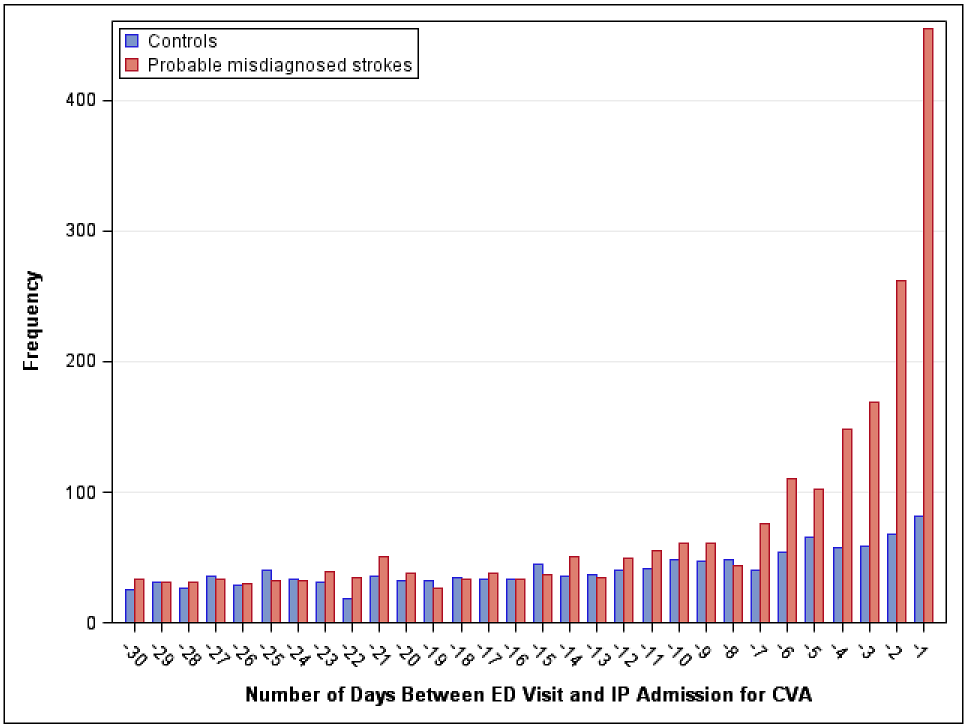

C. All other non-stroke, non-dizziness/headache, non-back/abdominal pain diagnoses (i.e., 'potential' misdiagnoses excluding correct diagnoses, probable misdiagnoses, and controls)

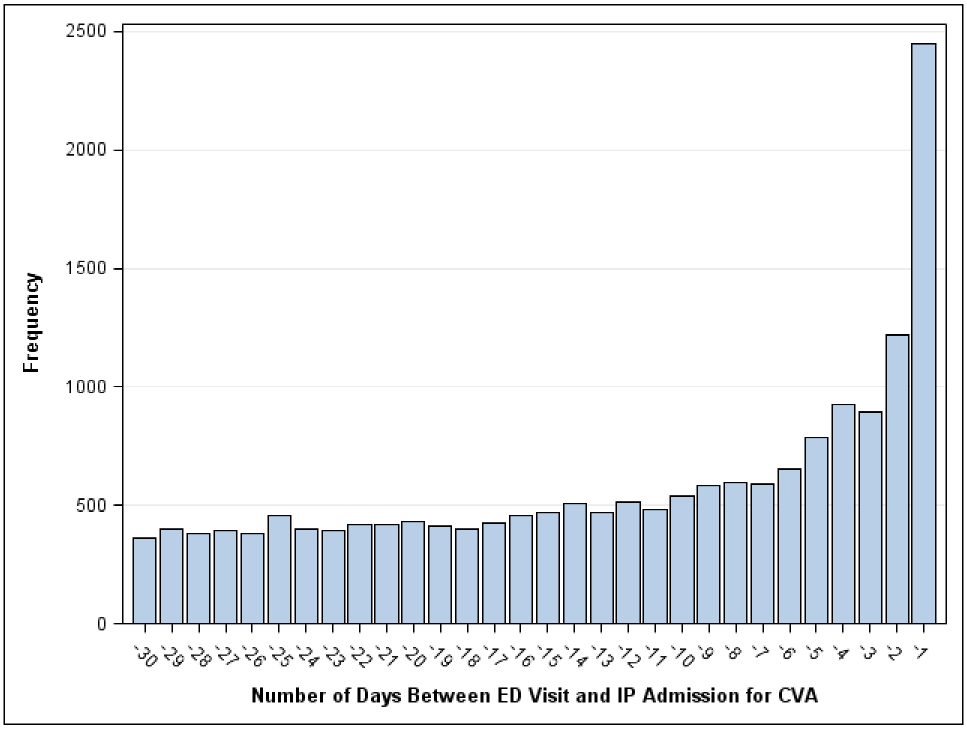

Supplement: Supplementary file 1 — Appendix 1 Table. Healthcare Cost and Utilization Project (HCUP) Clinical Classification System (CCS) Multilevel and ICD-9-CM codes used in analyses Appendix 2 Table. Observed emergency department (ED) non-adult (age <18 years) treat-and-release visits* that were followed by a stroke admission within 30 days, compared to the expected percentages, based on all ED treat-and-release visits Appendix 3 Figure. Temporal profile analysis of initial ED treat-and-release visits in the 30 days prior to an index stroke admission [file NIHMS851661-supplement-supplement_1.pdf]
